# Culturally sensitive stepped care for adolescent refugees: efficacy and cost–utility of a multicentric randomized controlled trial

**DOI:** 10.1007/s00787-023-02179-8

**Published:** 2023-03-16

**Authors:** Edgar Höhne, Kerem Böge, Carine Karnouk, Mira Tschorn, Tobias Banaschewski, Andreas Hoell, Thorsten Sukale, Paul Plener, Frank Schneider, Frank Padberg, Alkomiet Hasan, Michael A. Rapp, Malek Bajbouj, Inge Kamp-Becker

**Affiliations:** 1 Department of Child and Adolescent Psychiatry, Psychosomatics and Psychotherapy, Medical Clinic, Philipps-University Marburg, Marburg, Germany; 2 Department of Psychiatry and Psychotherapy, Campus Benjamin Franklin, Charité–Universitätsmedizin Berlin, Corporate Member of Freie Universität Berlin, Humboldt-Universität Zu Berlin, and Berlin Institute of Health, Berlin, Germany; 3 Department of Social and Preventive Medicine, University of Potsdam, Potsdam, Germany; 4 Department of Child and Adolescent Psychiatry and Psychotherapy, Medical Faculty Mannheim, Central Institute of Mental Health, Heidelberg University, Mannheim, Germany; 5 Department of Psychiatry and Psychotherapy, Medical Faculty Mannheim, Central Institute of Mental Health, Heidelberg University, Mannheim, Germany; 6 Department of Child and Adolescent Psychiatry and Psychotherapy, University of Ulm, Ulm, Germany; 7 Department of Child and Adolescent Psychiatry, Medical University of Vienna, Vienna, Austria; 8 University Hospital Düsseldorf, Medical School University of Duesseldorf, Düsseldorf, Germany; 9 Department of Psychiatry and Psychotherapy, University Hospital LMU, Munich, Germany; 10 Department of Psychiatry, Psychotherapy and Psychosomatics, Medical Faculty, University of Augsburg, BKH Augsburg, Augsburg, Germany

**Keywords:** Refugee minors, Depression, PTSD, Stepped care, Cultural sensitive

## Abstract

Adolescent refugees and asylum seekers (ARAS) are highly vulnerable to mental health problems. Stepped care models (SCM) and culturally sensitive therapies offer promising treatment approaches to effectively provide necessary medical and psychological support. To our knowledge, we were the first to investigate whether a culturally sensitive SCM will reduce symptoms of depression and PTSD in ARAS more effectively and efficiently than treatment as usual (TAU). We conducted a multicentric, randomized, controlled and rater-blinded trial across Germany with ARAS between the ages of 14 to 21 years. Participants (*N* = 158) were stratified by their level of depressive symptom severity and then equally randomized to either SCM or TAU. Depending on their severity level, SCM participants were allocated to tailored interventions. Symptom changes were assessed for depression (PHQ) and PTSD (CATS) at four time points, with the primary end point at post-intervention after 12 weeks. Based on an intention-to-treat sample, we used a linear mixed model approach for the main statistical analyses. Further evaluations included cost–utility analyses, sensitivity analyses, follow-up-analyses, response and remission rates and subgroup analysis. We found a significant reduction of PHQ (*d* = 0.52) and CATS (*d* = 0.27) scores in both groups. However, there was no significant difference between SCM and TAU. Cost–utility analyses indicated that SCM generated greater cost–utility when measured as quality-adjusted life years compared to TAU. Subgroup analysis revealed different effects for the SCM interventions depending on the outcome measure. Although culturally sensitive, SCMs did not prove to be more effective in symptom change and represent a more cost-effective treatment alternative for mentally burdened ARAS. Our research contributes to the optimization of clinical productivity and the improvement of therapeutic care for ARAS. Disorder-specific interventions should be further investigated.

**Supplementary Information:**

The online version contains supplementary material available at 10.1007/s00787-023-02179-8.

## Introduction

### Mental health of adolescent refugees and asylum seekers (ARAS)

There is vast evidence that ARAS are at a greater risk of developing psychological disorders than non-displaced children [1, 2]. Germany has accommodated the largest amount of ARAS in Europe in the past decade [3]. A recent meta-analysis from Germany reported high prevalence rates for PTSD (12.4–45.4%) and depression (30.6–39.8%) in minor refugees [4]. The comparably high heterogeneity within the prevalence rates can be attributed to varying survey periods, measures, study populations and risk and protective factors, such as stressful life events [5].

A meta-analysis regarding psychological interventions for minors after man-made or natural disasters reported medium effect sizes for symptoms of PTSD when compared with control conditions (Hedges’ *g* = 0.43) [6]. The most effective treatments for PTSD were cognitive behavioral therapy (CBT), Eye Movement Desensitization and Reprocessing (EMDR), Narrative Exposure Therapy for Children (KIDNET) and classroom-based interventions. For symptoms of depression, another meta-analysis on the effectiveness of psychological interventions in war-traumatized refugees reported a small mean pre–post effect for CBT treatment (Cohen’s *d* = 0.30) [7]. However, the access and success of psychological treatment has been shown to be very challenging for this population [8]. Patients with an immigration background show considerably low utilization rates of health support [9], higher rates of therapy discontinuations and poorer therapy outcomes [10]. Reasons for these barriers in therapeutic care can be divergent concepts of illness, language barriers, cultural misunderstandings or insufficient information about treatment options [11]. Culturally sensitive evidence-based interventions are a promising approach to face these challenges and are therefore, becoming more implemented in health care system [12–14]. But, despite the growing epidemiological relevance, culturally sensitive interventions have scarcely been investigated on adolescents so far [15]. In the course of the ongoing displacements and refugee movements across the world, the access to mental health services becomes even more crucial for young refugees [16]. To address this increasing demand for suitable mental health services, stepped and collaborative care models (SCM) might serve as a solution [17]. They appear to be more effective and resource friendly than usual care regarding the treatment of anxiety disorders and depression [18]. Within mental health, there are multiple ways of implementing SCMs. Some SCM designs use a progressive stepping-up approach [19], whereas others use stratified models which place patients depending on symptom severity [20]. Robust evidence has led to the recommendation of SCM by different international guidelines [21, 22]. Although SCM are apparently turning into a state-of-the-art treatment, they have not been investigated for the vulnerable population of young refugees so far [23].

### Aims of the study

We wanted to close this gap of research by investigating the effectiveness and efficiency of a culturally sensitive stratified SCM for ARAS experiencing symptoms of depression or PTSD. For this, we used data of four child and adolescent mental health clinics which took part in the “Mental Health in Refugees and Asylum Seekers (MEHIRA)” study, which investigated clinical and patient-reported outcomes as well as cost–utility of a stepped and collaborative care model for refugees in Germany [24]. Since the primary MEHIRA study did not differentiate between adolescent and adult refugees in their analyses [25], we explicitly wanted to investigate the effect of SCM on ARAS. According to the study protocol, we defined the following hypotheses: we assumed that a culturally sensitive SCM leads to a significant reduction of depressive symptoms between pre- and post-treatment compared to treatment as usual (TAU). Considering the high prevalence of PTSD within ARAS, we additionally aimed to investigate the effectiveness of the SCM on PTSD symptoms in comparison to TAU. Furthermore, we estimated that SCM will be superior to TAU in terms of costs per quality-adjusted life years. To gain more profound insight into individual effects of the adolescent-specific SCM levels, we performed an exploratory subgroup analysis for both outcome measures.

## Methods

### Study design

**Fig. 1:**
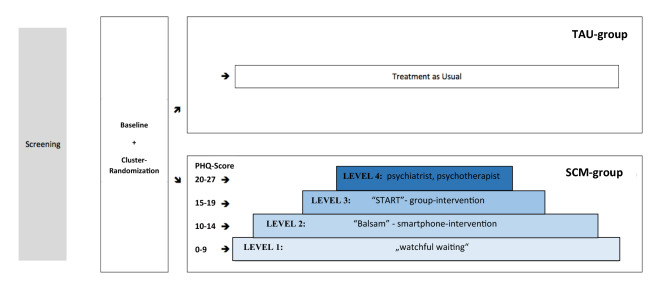
Illustration of study design and allocation process to SCM interventions

A longitudinal cluster-randomized controlled trial was conducted on a consecutive refugee sample with depressive symptoms in a rater-blinded setting at four university hospitals across Germany between May 2018 and March 2020. The stepped care approach included four interventions/levels: watchful waiting, smartphone application, group intervention and individual psychotherapy. Allocation to SCM interventions was based on depressive symptom severity (measured by PHQ-9). Intervention periods lasted 3 months for all levels. Figure 1 illustrates the study model and the allocation process to the SCM interventions. Sample size calculations and estimated dropout rates (50%) were only performed for the primary MEHIRA study. Detailed information of the study design (e.g., the randomization, blinding process, sample size calculations and data monitoring) are available in the study protocol [24]. The study design was not changed from the protocol. The study was conducted in accordance with the Declaration of Helsinki [26] and has been approved by the local ethics committees of all participating medical faculties. The study was registered in Clinical Trails.gov (registration number: NCT03109028).

### Recruitment

All study sites used diverse recruitment methods ranging from local school settings, outpatient settings as well as refugee housing and accommodation centers. Study teams presented the study and distributed flyers about the study in the mentioned locations to promote recruitment. No financial compensation was offered. For study inclusion, participants had to be asylum seekers or refugees according to the office of the United Nations High Commissioner for Refugees (UNHCR) definition [27], 14–21 years of age with sufficient language skills in Arabic, Farsi/Dari, English or German. Further, they had to show at least mild depressive symptoms (≥ 5 in the PHQ-9/PHQ-A) and psychological distress (≥ 12 for the items 1–14 or ≥ 5 for item 15 in the Refugee Health Screener (RHS)-15) at screening assessment (T-1). Exclusion criteria were symptoms of a psychotic or degenerative disorder and/or an acute risk of suicidality (≥ 4 on item 10 in the Montgomery–Åsberg Depression Rating Scale—MADRS) [28]. Prior to study begin, all participants were informed about study content, objectives and anonymous processing of the data and gave written informed consent. For participants aged 14–17 years, a written consent from their legal guardian was obtained.

### Study procedure

Before the initial trial onset, all participants underwent a screening phase within 4 weeks to 1 day prior to randomization, where affective symptom severity was assessed (T-1). All screenings were conducted by trained psychologists to ensure consistency. After the screening process, participants were cluster randomized to either SCM or TAU at baseline (T0). Randomization was performed by an independent coordinating center for clinical trials in a 1:1 ratio. At baseline, symptomatology and socioeconomic information were assessed by trained psychotherapists via questionnaire and clinical interview. In the next step, participants received either a symptom-tailored SCM intervention or treatment as usual (TAU) for the next 12 weeks, directly followed by post-treatment assessment (T1). Follow-up measures were administered 12 weeks (T2) and 24 weeks (T3) after post-treatment assessment with predefined maximum deviation of 3 weeks.

### Interventions

According to development guidelines for culturally sensitive interventions, people with a refugee background contributed to the development of the interventions as advisors, and focus group members as well as within the study in the role of translators and research assistants [29]. The distribution of baseline PHQ scores was used to assign the patients to suitable groups of the SCM. Participants were allocated to one of the following treatment levels according to validated severity classification [30]:

#### Watchful waiting (level 1: PHQ score 5–9)

Participants with mild depressive symptoms did not receive an active intervention, but contact details were provided in case mental support was needed.

#### Smartphone application “Balsam” (level 2: PHQ score 10–14)

Participants with moderate depressive symptoms received a smartphone application “Balsam”. This app was recently developed in the context of the MEHIRA project by a group of psychologists and researchers from diverse cultural backgrounds to “help migrants and refugees understand the underlying mechanisms of their stress and arm themselves with the appropriate tools to cope with their struggles” [24]. It contains more than 80 videos, is available in 4 languages (Arabic, Farsi, English and German) and includes 15 different modules covering psychoeducative elements, therapeutic exercises, built-in questionnaires and an emergency support function. Participants were encouraged to use the app daily via push notifications.

#### Group intervention “START_adapt” (level 3: PHQ score 15–19)

Participants with moderate–severe depressive symptoms were assigned to the “START_adapt” group intervention. This intervention was adapted from the Stress-Traumasymptoms-Arousal-Regulation-Treatment (START), a short cultural sensitive group intervention for adolescents affected by severe trauma and extreme emotional stress [31]. It was designed as a brief and standardized five-session therapeutic program for four to eight refugee minors based on elements of dialectical-behavior therapy (DBT) and trauma-focused cognitive behavioral therapy (TF-CBT). Groups were led by a licensed psychotherapist and a trained assistant. Instructions and information were available in four languages (Arabic, Farsi, English and German) and multiple visual displays were included in the manual. To allow for multi-lingual groups without interpreters, all contents were available by audio via an mp3-player.

#### Psychotherapy (level 4: PHQ score 20–27)

Participants with severe depressive symptoms received an individual psychological treatment by a licensed psychotherapist. The non-manualized treatment was based on cognitive behavioral approaches. If needed, a translator was provided.

#### Treatment as usual: TAU

Participants randomized to TAU were allowed to receive all available routine health-care services regardless of symptom severity. Participants were not guided and had to seek help to relieve the symptoms on their own. There were no regulations concerning the institutions, operators or kind of treatments participants received.

### Measures

Self-rated questionnaires were available in validated Arabic, Farsi, English and German versions. Rater-based assessments were performed by a trained psychiatrist or psychologist who was blinded to the trial condition, if needed with the assistance of a translator. A comprehensive list of all measures can be found in the study protocol [24]. The following two instruments were used as measures for the clinical outcomes:

#### Patient Health Questionnaire (PHQ-9/PHQ-A)

The primary outcome, depression symptom severity, was assessed via age-related versions of the PHQ-9. The PHQ-9 is a brief and widely used nine-item self-report instrument based on DSM-IV to assess the frequency of depressive symptoms within the last 2 weeks on a four-point Likert scale [30]. The sum score can range from 0 to 27. Internal consistency of the PHQ-9 is high, with a Cronbach’s alpha of 0.86–0.89 [source]. Adolescents under the age of 18 years filled out the PHQ-A, a slightly modified, highly comparable and well-validated version of the PHQ-9 for adolescents [32].

#### Child and Adolescent Trauma Screen (CATS)

The secondary outcome was assessed via CATS, a short self-report instrument based on DSM-V to assess PTSD symptoms in children and adolescents [33]. Symptoms are assessed on a four-point Likert scale and can range from 0 to 60. Internal consistency of the symptom screening is high, with a Cronbach’s alpha of 0.88–0.94 [33].

#### Resource use and costs

Resource use was measured with an adapted version of the Mannheim-Module-Resource-Use (MRV) [35]. We performed the MRV as interview by trained staff from T0 to T3 and asked for the utilization of inpatient and outpatient health services, remedies, counseling and health support services, and medication. Data on resource use were then combined with unit costs. Unit costs were derived from nationally or regionally available data sources. We adopted a health-care system perspective including direct interventions costs as well. We calculated per patient costs for the SCM as a combination of a) recurring or running expenses due to personnel and operating costs and b) one-off costs defined as costs of development for each intervention including tutorial sessions, and preparation of manuals. Costs of SCM were derived via interviews with key persons of depression symptom severity-specific intervention types. We adjusted all prices to the reference year 2019 in euros, but did not discount costs due to the short time horizon of the study.

#### Determination of utilities

As economic end point for the cost–utility analysis, we considered cost per quality-adjusted life year (QALY). We derived QALYs from the abbreviated World Health Organization Quality of Life questionnaire (WHOQOL-BREF) from T0 to T3 [34] using the method proposed by Salize and Kilian [35] and extrapolated values for a 12-month period.

### Statistical analysis

Data analysis was performed using IBM SPSS statistics version 22, SAS version 9.4 (SAS Institute Inc., Cary, North Carolina, USA) and Excel 2016 for Windows. The intention-to-treat (ITT) sample included all randomized participants who provided baseline data of the PHQ. For sensitivity analysis, the per protocol (PP) sample excluded all participants who dropped out at T1, showed missing data at T1, participated in < 50% of the intervention sessions or did not receive the allocated intervention. Differences in sample characteristics between SCM and TAU at baseline were investigated using ANOVA for continuous outcomes, and chi-square tests for categorical variables. In case of significant differences, variables were included as covariates in the main analysis. For the main analyses, we calculated a linear mixed model analysis (LMM) using the ITT sample with time (T0–T1) and group*time–interaction as fixed effects. Intraclass correlation coefficients (ICC) indicated that a nontrivial proportion of total variance was attributed to the study centers (0.12–0.32). Thus, we added study center as random effects to our LMM analyses. To check for robustness of findings, we performed the analyses for the PP sample (sensitivity analysis) and for all time points (follow-up analyses). Tests were two tailed and statistical significance was set at a *p* value < 0.05. If significant, effect sizes were calculated [36]. To evaluate the significance of change for the individual, response (≥ 50% PHQ-9 reduction at T1) and remission (PHQ-9 < 5 points at T1) rates were calculated for all patients with post-intervention PHQ scores.

Statistical analyses for health-care costs and cost–utility were performed using the ITT sample (base case-scenario). Missing values from T1 to T3 were replaced using the last observation carried forward (LOCF) approach. We applied generalized linear models (GLM) with gamma distributions and identity link function to estimate differences in log-transformed health-care costs between SCM and TAU. We determined the incremental cost-effectiveness ratios (ICER) which represent the additional costs to obtain one additional QALY. The ICER was calculated as the ratio between the differences in mean costs and the differences in mean QALY between SCM and TAU. We considered statistical uncertainty around the point estimate with nonparametric bootstrapping with 10,000 samples. In addition, we performed net monetary benefit (NMB) analyses to consider the likelihood of cost–utility against different willingness to pay thresholds. To check for robustness of findings, we performed sensitivity analyses. Therefore, we varied the conditions of calculating per capita costs of SCM by a) evaluating average costs on the basis of the total number of theoretically assigned participants considering that all intervention types had an expected capacity utilization of 100% (optimal scenario) and b) excluding resource use costs and considering intervention costs alone (on-top scenario).

## Results

### Recruitment and sample characteristics

**Fig. 2:**
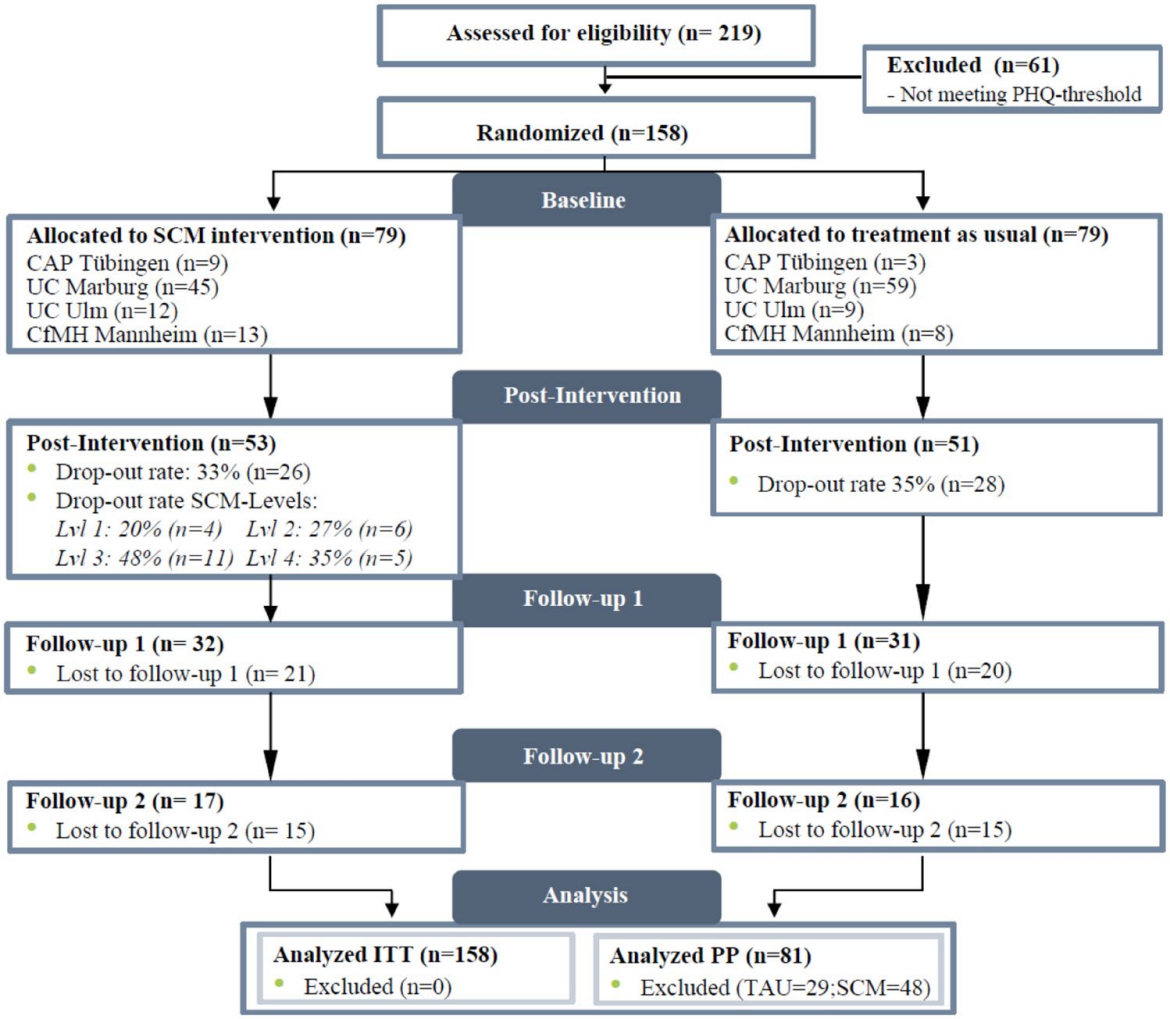
Flow of participants according to cluster-randomized CONSORT statement

The flow of participants is presented in Fig. 2. A total of 219 ARAS were interested in participation and gave informed consent. Since 61 participants did not exceed the PHQ threshold for inclusion, the ITT sample included 158 participants (*n* = 79 SCM, *n* = 79 TAU) with *n* = 20 in level 1, *n* = 22 in level 2, *n* = 23 in level 3 and *n* = 14 in level 4 of the SCM. Post-intervention assessments were completed by 104 participants (*n* = 53 SCM, *n* = 51 TAU), leaving a total dropout rate (= missing data at T1) of 34% with similar dropout rates for SCM and TAU. In level 3 of the SCM, dropout rates were considerably high (48%) compared to the other levels. The majority of the participants were male (84.18%), with a mean age of 18.06 years (SD 1.58), and immigrated from Afghanistan (29.10%), Syria (23.13%), Iran (12.69%) or Eritrea (6.72%). Sample characteristics were representative for the ARAS in Germany (BAMF, 2019). For detailed baseline sample characteristics see Table 1. There were no significant baseline differences regarding sample characteristics and outcome measures in the ITT sample. In the PP sample, we found a significant difference in baseline CATS score between SCM and TAU (*p* < 0.05). We adjusted this imbalance by adding the covariate T0-CATS score to the LMM analysis. No serious adverse effects were reported for both treatment arms [37].

**Table 1:** Baseline sample characteristics on total ITT, SCM and TAU sample

	Mean ± SD; N/total *N* (%)			*P* value
	ITT (*N* = 158)	SCM (*n* = 79)	TAU (*n* = 79)	
Age (years)	18.06 ± 1.58	17.94 ± 1.62	18.18 ± 1.53	0.34
Female	25/158 (15.82)	12/79 (15.19)	13/79(16.45)	0.83
Date of immigration	05/14/2016	05/31/2016	04/27/2016	0.66
Years of education	7.08 ± 3.22	6.86 ± 3.07	7.34 ± 3.38	0.39
PHQ value	14.79 ± 72	14.48 (0.82)	15.12 ± 0.87	0.47
CATS value	30.95 ± 2.13	29.89 ± 2.27	32.00 ± 2.46	0.31
WHOQOL-BREF value	56.70 ± 13.03	55.82 ± 13.91	57.67 ± 12.02	0.41
QALY value		0.48 ± 0.22	0.46 ± 0.22	0.57
Country of origin				0.73
Afghanistan	39/134 (29.10)	21/71 (29.58)	18/63 (28.57)	
Syria	31/134 (23.13)	18/71 (25.35)	13/63 (20.64)	
Iran	17/134 (12.69)	7/71 (9.86)	10/63 (15.87)	
Eritrea	9/134 (6.72)	4/71 (5.63)	5/63 (7.94)	
Other (< 5%)	38/134 (28.36)	21/71 (29.58)	17/63 (26.98)	
Current accommodation				0.64
Private flat	34/131 (25.95)	18/70 (25.71)	16/61 (26.23)	
Refugee accommodation	58/131 (44.27)	32/70 (45.71)	26/61 (42.62)	
Shared flat	29/131 (22.14)	13/70 (18.58)	16/61 (26.23)	
Assisted living	10/131 (7.63)	7/70 (10.00)	3/61 (4.92)	
Current state of residence				0.31
Permanent permit	9/134 (6.72)	3/71 (4.23)	6/63 (9.52)	
Temporary permit	111/134 (82.83)	59/71 (83.09)	52/63 (82.54)	
No legal permit	3/134 (2.24)	1/71 (1.41)	2/63 (3.18)	
Other	11/134 (8.21)	8/71 (11.27)	3/63 (4.76)	
Reason for migration^a^				
War	79/143 (55.24)	41/76 (53.95)	38/67 (56.72)	0.74
Natural disaster	2/143 (1.39)	1/76 (1.31)	1/67 (1.49)	0.93
Economic crisis	15/143 (10.49)	11/76 (14.47)	4/67 (5.97)	0.10
Individual situation	24/143 (16.78)	13/76 (17.10)	11/67 (16.42)	0.91
Political/religious persecution	67/143 (46.85)	34/76 (44.74)	33/67 (49.25)	0.59
Social situation	28/143 (19.58)	17/76 (22.37)	11/67 (16.42)	0.37
Other	31/143 (21.68)	17/76 (22.37)	14/67 (20.89)	0.83

*PHQ-9* Patient Health Questionnaire, 9-item module, *CATS* Child and Adolescent Trauma Screen, *WHOQOL-BREF* World Health Organization Quality of Life Brief version, *QALY* quality-adjusted life year

For participants allocated to TAU, resource usage data was available for *n* = 49 between T0 and T1. Most TAU participants (63%) sought medical support with an average of 2.32 appointments (SD 1.77). One-third (33%) sought psychiatric/psychological support with an average of 4.07 appointments (SD 3.34). 43% of the TAU participants received a medication via pharmacy. One participant of the TAU sample stayed at a psychiatric clinic for 6 days and another one stayed at a hospital because of medical reasons for 21 days.

### Primary analysis

Results of the primary analysis are shown in Table 2. The analysis of PHQ data showed a significant adjusted mean reduction for both treatment groups from T0 to T1 in the ITT sample (*F*_1,120_ = 26.92, *p* < 0.0001, *d* = 0.524) and PP sample (*F*_1,81_ = 21.07, *p* < 0.0001; *d* = 0.494). SCM and TAU did not significantly differ in symptom reduction from T0 to T1 in the ITT sample (*F*_2,150_ = 0.27, *p* = 0.762) and PP sample (*F*_2,80_ = 1.97, *p* = 0.146). Follow-up analysis showed a significant adjusted mean reduction from T0 to T3 for both treatment groups in the ITT sample (*F*_3,76_ = 6.61, *p* < 0.0001; *d* = 0.496). Again, SCM and TAU did not significantly differ in symptom reduction (*F*_4,63_ = 0.20, *p* = 0.937). Response rates were 26.4% in the SCM and 23.5% in the TAU group (i.e., ≥ 50% reduction of PHQ-9 score) to their treatment from T0 to T1. Remission rates were 17% in the SCM and 15.7% in the TAU condition (i.e., PHQ-9 score < 5 points) of symptoms of depression at T1. SCM and TAU did not differ significantly in terms of response and remission rates (*p* > 0.80). Results of response and remission analysis are shown in Table 3.

**Table 2:** Results of primary and secondary outcome analysis, sensitivity analysis and follow-up analysis

	Total *N*			SCM			TAU			∆SCM /TAU (*p*)	Effect of time (*p*)	Effect of group*time (*p*)
	Mean (SE)	Adjusted mean change (95% CI)	∆ T0 (*p*)	Mean (SE)	Adjusted mean change (95% CI)	∆ T0 (*p*)	Mean (SE)	Adjusted mean change (95% CI)	∆ T0 (*p*)			
Primary outcome analysis												
Depression severity from T0 to T1 in the ITT sample (assessed by PHQ)												
T0	14.79 (0.72)			14.48 (.82)			15.12 (.87)			0.468		
T1	12.11 (0.78)	− 2.68 (− 3.71; − 1.66)	< 0.0001	11.89 (.91)	− 2.58 (− 4.02;− 1.15)	< 0.001	12.34 (.96)	− 2.78 (− 4.24; − 1.32)	< 0.001	0.668	< 0.0001	0.762
PTSD severity from T0 to T1 in the ITT sample (assessed by CATS)												
T0	30.95 (2.13)			29.89 (2.27)			32.00 (2.46)			0.307		
T1	27.63 (2.17)	− 3.32 (− 5.47;− 1.16)	< 0.005	28.42 (2.37)	− 1.48 (− 4.49; 1.54)	0.334	26.85 (2.53)	− 5.16 (− 8.24; − 2.08)	< 0.001	0.488	< 0.005	0.230
Sensitivity analysis												
Depression severity from T0 to T1 in the PP sample (assessed by PHQ)												
T0	13.75 (1.50)			12.95 (1.64)			14.54 (1.60)			0.201		
T1	10.89 (1.50)	− 2.85 (− 4.08; − 1.61)	< 0.0001	9.69 (1.64)	− 3.26 (− 5.19; − 1.32)	< 0.001	12.10 (1.60)	− 2.44 (− 3.96; − 0.91)	< 0.005	0.053	< 0.0001	0.146
PTSD severity from T0 to T1 in the PP sample (assessed by CATS)												
T0	28.58 (3.78)			25.21 (4.04)			31.94 (3.97)			< .05		
T1	26.24 (3.78)	− 2.34 (− 4.98; 0.30)	0.082	25.53 (4.04)	0.32 (− 3.84; 4.47)	0.880	26.95 (3.97)	− 4.99 (− 8.27;− 1.73)	< 0.005	0.598	0.082	0.125
Follow-up analysis												
Depression severity from T0 to T3 in the ITT sample (assessed by PHQ)												
T0	14.61 (0.95)			14.32 (1.02)			14.89 (1.05)			0.506		
T1	11.76 (1.02)	− 2.85 (− 4.69;− 0.99)	< 0.0001	11.62 (1.13)	− 2.70 (− 5.29;− 0.10)	< 0.05	11.90 (1.18)	− 2.99 (− 5.62;− 0.36)	< 0.05	0.800		
T2	12.19 (1.15)	− 2.41 (− 4.72; − 0.10)	< 0.05	12.27 (1.34)	− 2.06 (− 5.29; 1.18)	0.543	12.13 (1.39)	− 2.77 (− 6.04; 0.50)	0.149	0.924		
T3	12.44 (1.53)	− 2.17 (− 5.79; 1.46)	0.63	11.78 (1.94)	− 2.54 (− 7.60; 2.52)	1.00	13.09 (1.99)	− 1.79 (− 6.97; 3.38)	1.00	0.595	<0 .0001	0.937
PTSD severity from T0 to T3 in the PP sample (assessed by CATS)												
T0	30.87 (1.95)			29.84 (2.11)			31.91 (2.30)			0.318		
T1	27.59 (1.99)	− 3.28 (− 6.04; − 0.51)	< 0.05	28.45 (2.21)	− 1.39 (− 5.27; 2.48)	1.00	26.75 (2.37)	− 5.16 (− 9.11;− 1.21)	< 0.005	0.448		
T2	27.20 (2.19)	− 3.67 (− 7.66; 0.31)	0.090	26.81 (2.53)	− 3.03 (− 8.62; 2.57)	0.911	27.59 (2.65)	− 4.32 (− 9.97; 1.34)	0.261	0.778		
T3	29.06 (2.58)	− 1.81 (− 7.22; 3.59)	1.00	29.78 (3.12)	− 0.06 (− 7.59; 7.47)	1.00	28.35 (3.24)	− 3.56 (− 11.29; 4.17)	1.00	0.701	< 0.05	0.406

**Table 3:** Results of primary outcome analysis and sensitivity

	SCM		TAU		*p*
	*n*	%	*n*	%	
Response	14	26,4%	12	23,5%	0.822
Remission	9	17,0%	8	15,7%	1.00

Response ≥ 50% reduction of PHQ-9 score from baseline to T1. Remission = a PHQ-9 score < 5 points at T1

### Secondary analysis

Results of the secondary analysis are shown in Table 2. The analysis of CATS data in the ITT sample showed a significant adjusted mean reduction for both treatment groups from T0 to T1 (*F*_1,115_ = 9.31, *p* < 0.005; *d* = 0.274) in the ITT sample, but not in the PP sample (*F*_1,81_ = 3.10, *p* = 0.082). Again, SCM and TAU did not significantly differ in symptom reduction from T0 to T1 in the ITT sample (*F*_2,127_ = 1.48, *p* = 0.230) and PP sample (*F*_2,79_ = 2.14, *p* = 0.125). The follow-up analysis showed a significant adjusted mean reduction for both groups from T0 to T3 (*F*_3,222_ = 3.81, *p* < 0.05; *d* = 0.243). Again, SCM and TAU did not significantly differ in symptom reduction (*F*_4,235_ = 1.01, *p* = 0.406).

### Subgroup analysis

The analysis of PHQ data showed a significant adjusted mean reduction for all levels combined from baseline to post-intervention (*F*_1,77_ = 19.77, *p* < 0.0001). The SCM interventions significantly differed in symptom reduction from T0 to T1 (*F*_6,119_ = 30.34, *p* < 0.0001). Only level 4 (psychotherapy) showed a significant mean difference from T0 to T1 (*F*_1,78_ = 24.04, *p* < 0.0001). Level 3 (group-therapy) trended toward a significant mean difference (*F*_1,85_ = 3.79, *p* = 0.055). The analysis of CATS data showed no significant adjusted mean reduction for all levels combined from baseline to post-intervention (*F*_1,60_ = 0.38, *p* = 0.541). The SCM interventions significantly differed in symptom reduction from T0 to T1 (*F*_6,69_ = 9.45, *p* < 0.0001). Only level 2 (smartphone-app) showed a significant mean difference from T0 to T1 (*F*_1,60_ = 4.97, *p* < 0.05).

### Health-care costs

Health-care costs and resource use data were available for 139 participants (SCM = 74; TAU = 65). One year after baseline, SCM and TAU did not differ significantly in terms of average resource use costs (*p* = 0.884) and total costs including SCM program costs (*p* = 0.096). The largest component of the total costs was psychological/psychiatric support for both groups. Results of per capita cost for each intervention are shown in Table 4.

**Table 4:** Comparison of per capita costs of SCM vs. TAU (base case)

	SCM (*n* = 74) mean (SD) in €	TAU (*N* = 65) mean (SD) IN €	*P*
Emergency			
Primary care incl language mediator	180.1 (156.7)	191.5 (153.3)	
Psychiatric, psychological support	498.9 (1,174.8)	591.5 (1,478.2)	
General hospital	492.2 (1,778.7)	119.7 (480.2)	
Other therapies (outpatient)	16.4 (65.1)	1.7 (13.4)	
Medication	169.1 (275.5)	207.2 (246.3)	
Resource use costs	1,423.2 (2,201.4)	1,143.5 (1,590.8)	0.884
Program costs (recurrent personnel and operating costs)	218.9	0	
Total costs	1,642.1 (2,201.4)	1,143.5 (1,590.8)	0.096

### Cost–utility analyses

For the cost–utility analyses, data were available for 139 participants (SCM = 74; TAU = 65). Results of the incremental costs, utilities (QALY) and ICER of SCM compared to TAU are shown in Table 5. After 1 year, QALYs increased in SCM compared to TAU (Mean difference = 0.154; 95; CI   − 0.260 to 0.474, *p* = 0.450). The ICER for an additional QALY in the SCM base case scenario compared to TAU was calculated on the basis of incremental costs of €468.9 (recurrent costs) and incremental utility value of 0.154, which led to an ICER of 3138€ (95% CI   -33,488—35,518€). The ICER for an additional QALY in the SCM optimal case scenario was 2,842€ (95%CI − 33,452–35,606€). The distribution of boot-strapped ICER on the cost–utility plane for SCM base case is shown in Table 6. The distribution revealed that the majority of ICER were located in the north-east quadrant (base case 66.5%; optimal case 64.8%). The distribution indicates that SCM is more expensive and produces more QALYs than TAU at the same time. The net monetary benefit analysis (NMB) for SCM base case with recurrent costs showed a probability of 5.8% for SCM to obtain an additional QALY without any additional costs. Concerning different WTP thresholds, SCM dominates TAU regarding additional costs per QALY at values above €5,000.

**Table 5:** Results of incremental costs, effects (QALY) and ICER of SCM compared to TAU for all scenarios

	Mean	95% CI
Base case		
Incremental costs (recurrent)	468.9€	(− 121.6 to 1058.4€)
Incremental QALY	0.154	(− 260 to 0.474)
ICER (€/QALY)	3,138.1€	(− 33,488.8 to 35,518.3€)
Optimal case		
Incremental costs (recurrent)	424.7€	(− 175.6 to 1,032.2€)
Incremental QALY	0.154	(− 260 to .474)
ICER (€/QALY)	2,842.2€	(− 33,452.2 to 35,606.0€)
On-top case		
Incremental costs (recurrent)	218.9€	
Incremental QALY	0.154	(− 260 to 0.474)
ICER (€/QALY)	1,465.0€	(− 15,926.9 to 15,643.2€)

*QALY* quality-adjusted life year, *ICER* incremental cost-effectiveness ratio

**Table 6:** Cost–utility analyses and net monetary benefit

	Cost–utility on patient-reported outcome: QALY		
	Base case	Optimal case	On-top case
Distribution of boot-strapped ICER on CU plane (in percent)			
First quadrant (north-east)	66.5	64.8	70.2
Second quadrant (inferior: north-west)	27.7	26.8	29.8
Third quadrant (south-west)	1.4	2.0	0.0
Fourth quadrant (dominant: south-east)	4.4	6.4	0.0
Probability of cost–utility according to predefined WTP-thresholds (in percent)			
€0	5.8	8.4	0
€1,000	16.0	18.8	25.9
€5,000	51.9	53.5	61.7
€10,000	61.4	62.6	66.2
€20,000	66.5	66.9	68.4
€50,000	69.2	69.7	69.5
€100,000	70.0	70.5	70.2

Comparison between SCM and TAU on distribution of ICER on cost–utility plane and on probability of cost–utility according to predefined willingness to pay thresholds

*ICER* incremental cost-effectiveness ratio, *CU* cost–utility, *QALY* quality-adjusted life year, *WTP* willingness to pay

## Discussion

Our study aimed to investigate the effectiveness and efficiency of a culturally sensitive SCM for ARAS with mental health problems. Compliance with the study (dropout rate 34%) was more satisfying than previously expected [24]. The results of the primary and secondary analyses showed a significant reduction of clinical outcomes in ARAS for both study arms after 12 weeks, with moderate effect sizes for depressive symptoms and small effect sizes for PTSD symptoms. Effects were stable over time for both clinical outcomes. Sensitivity analysis showed robustness of effects for the depression outcome. However, SCM patients did not show a superior clinical outcome compared to TAU, in PHQ or CATS measures. Although SCM and TAU did not significantly differ in terms of overall health-care costs, costs needed to increase utility values in the forms of QALYs for ARAS were smaller in SCM. Thus, in line with existing SCM analyses in adults [18], SCM is cost-effective compared to routine mental health care for ARAS. This result is crucial in face of financial restrictions and rational allocations of resources in health-care systems worldwide.

In contrast to our hypothesis and to existing evidence for anxiety and depression in non-migrant adults [20], SCM was not superior to TAU in terms of symptom reduction. Nonetheless, within the overall MEHIRA study, including both adult and adolescent refugees, SCM significantly reduced depressive symptoms compared to TAU [25]. A reason for the missing differences between SCM and TAU in terms of symptom change in this study might be that the TAU group made use of a considerable amount of support by health authorities and other caretakers. For example, TAU participants used a similar amount of (mental) health-care resources during the observation period as the SCM participants, which led to insignificantly different overall per capita health-care costs between groups. Here, the intervention costs, costs that are borne by the SCM group alone, are included. Also, it is likely that the application of SCM for ARAS would show stronger effects on symptomatology in low-income countries with less secure welfare systems. This assumption is supported by two stepped care RCTs from low-income countries. One Sudanese RCT reported a significantly higher symptom change of SCM compared to TAU [38], whereas a Nigerian RCT reported no differences between an enhanced TAU and SCM [39]**.**

Further, as indicated by our subgroup analyses, the effectiveness of the individual SCM levels varied considerably depending on the clinical outcomes which might also explain why our hypothesis was not supported by the results. This is in line with a systematic review on stepped care for the treatment of depression, which reported a high heterogeneity between the different SCM approaches [40]. For symptoms of depression, the effect of our SCM was mainly driven by the interventions from level 3 and 4, whereas for PTSD symptoms, especially, the group intervention curbed the overall effect of the SCM (see supplements). This is in line with an Australian study, which also found improvements of depressive, but not PTSD symptoms in refugee children by group CBT [41]. Further, a meta-analysis on psychosocial interventions for traumatized children and adolescents reported significantly lower effect sizes if treatment was delivered in a group setting [6]. However, another study with a notably larger sample size for investigating group-treatment (*n* = 43 vs. *n* = 14), reported improvements of PTSD and depression symptoms [42]. In our study, the recruitment of a sufficient amount of participants for a group intervention was limited due to the narrowly defined time and symptom window (4 weeks, PHQ-score 15–19). As a result, participants allocated to the group intervention had to be declared as dropouts when the sufficient group size was not reached in the predefined time window. This also explains the disproportionally high dropout rates for this intervention (48%). In addition, some ARAS refused to take part in a group intervention. They reported the large spectrum of age, the different cultural backgrounds, the mixed-gender group design and fears of stigmatization by others as reasons for their reluctance. Similarly, another intervention study showed country of origin to be a significant predictor of symptom improvement in a group intervention [42]. They reported a higher mean change on the CATS score for ARAS from African countries compared to ARAS from countries in the Middle East. Participants in our group intervention mainly emigrated from the Middle East. Future research of group interventions for ARAS should investigate differential treatment moderators (e.g., similar age, cultural background, accommodation status) and define broader time slots for recruitment.

In contrast to the group intervention, the smartphone intervention (level 2) significantly reduced symptoms of PTSD. These findings are supported by results of other low-intensity psychological interventions [43, 44]. The digital aspect of this intervention may have helped ARAS, to whom smartphone applications represent a very familiar medium, to overcome the earlier mentioned language barriers and fear of stigmatization. Since patients with low PTSD severity tend to improve less by psychological treatments [45], smartphone applications can serve as a promising treatment alternative [46]. However, in line with the primary MEHIRA study [25], the smartphone intervention did not improve mild depressive symptoms. Similar observations were reported in a meta-analysis, which found a tendency for reduced effects of low-intensity intervention on mild depressive symptoms [47]. Future research should further investigate the influence of initial symptom severity on the effectiveness of digital interventions in ARAS.

### Strengths and limitations

A major limitation of this study is the lack of specific inclusion criteria for PTSD symptomatology, which was only assessed broadly via the RHS-15. This recruitment strategy introduces bias into this study and the PTSD results must be interpreted with caution. However, the exploratory subgroup analysis revealed no critical discrepancies for severity distribution of symptoms of PTSD in the SCM levels (see supplements). The results of the exploratory subgroup analysis must be critically reviewed in light of the considerably small sample sizes for the single intervention levels, and thus be interpreted primarily as an insight into the driving forces of the SCM. Another limitation of this study is the incomplete evaluation of potential moderators for mental health outcomes such as accompaniment, language skills or perceived social support.

A strength of this randomized controlled trial lies in its large and representative sample being examined at four different time points in multiple study sites. Further, with the cost–utility analysis, our study results go beyond just symptom improvement. Moreover, our newly developed culturally sensitive SCM interventions were implemented and investigated in ARAS for the first time. Results are not only relevant to the German, or European context, but can also be expanded to broader international contexts.

## Conclusion

To the best of our knowledge, this is the first RCT to provide evidence for the effectiveness of a culturally sensitive SCM treatment approach in ARAS. Even though SCM and TAU demonstrated similar effects on symptom change, cost analyses suggest that SCM are superior to TAU in terms of cost–utility. Our research contributes to the optimization of clinical productivity in a currently tense health-care situation and shows possibilities to improve resource allocations. Further, our investigation of novel culturally sensitive interventions depicts an important contribution to the early prevention of mental health problems and improvement of therapeutic care for ARAS. Especially, the newly developed smartphone intervention can be very useful in contexts where direct care and resources are not always available, such as crisis regions and low-income countries. Our exploratory subgroup analysis provides first ideas for future research on how to further improve this newly developed SCM. Differential effects regarding symptoms of depression and PTSD point toward the urgent need for disorder-specific treatments of ARAS.

### Supplementary Information

Below is the link to the electronic supplementary material.

Supplementary file1 (DOCX 40 KB)

## Data Availability

The data that support the findings of this study are available from the corresponding author, [EH], upon reasonable request.

## Abbreviations

PTSD: Posttraumatic stress disorder
PHQ: Patients Health Questionnaire
CATS: Child and Adolescent Trauma Screen
