# Supplementary material for: Culturally sensitive stepped care for adolescent refugees: efficacy and cost–utility of a multicentric randomized controlled trial
Source: Eur Child Adolesc Psychiatry. 2023 Mar 16;33(2):581–93. doi: 10.1007/s00787-023-02179-8 (PMC10869413; doi:10.1007/s00787-023-02179-8)

**Supplemental A: Subgroup-Analysis for Depression-severity^a^**

**Tests for fixed effects, Typ IIIa**

| Source | dfn | dfd | F | Sig. |
| --- | --- | --- | --- | --- |
| Konstanter Term | 1 | 1,381 | 356,672 | ,012 |
| time | 1 | 77,296 | 19,765 | **,000** |
| time * SCCM Level | 6 | 119,623 | 30,335 | **,000** |
| a. outcome measure: PHQ_Score, dfn = degrees of freedom numerator, dfd degrees of freedom denominator | | | | |

**Estimated marginal means**

| SCCM Level |  |  | Mean difference (T0-T1) | Standarderror (SE) | degrees of freedom (df) | significance (p) | 95% CI for the mean difference | |
| --- | --- | --- | --- | --- | --- | --- | --- | --- |
|  |  |  |  |  |  |  | Lower limit | Upper limit |
| Level 1: | | | -,240 | 1,156 | 70,306 | ,836 | -2,545 | 2,066 |
| Level 2: | | | -1,285 | 1,136 | 73,583 | ,261 | -3,548 | ,978 |
| Level 3: | | | -2,409 | 1,238 | 85,606 | ,055 | -4,871 | ,053 |
| Level 4: | | | -7,257* | 1,480 | 78,040 | **,000** | -10,204 | -4,311 |


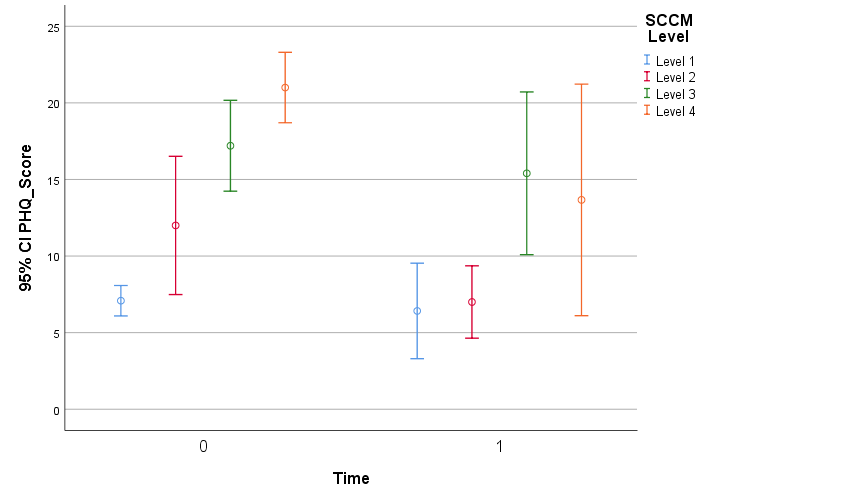


**Supplemental B: Subgroup-Analysis for PTSD-severity^a^**

**Tests for fixed effects, Typ III^a^**

| Source | dfn | dfd | F | Sig. |
| --- | --- | --- | --- | --- |
| Konstanter Term | 1 | 1,647 | 191,663 | ,011 |
| time | 1 | 60,782 | ,378 | ,541 |
| time * SCCM Level | 6 | 69,276 | 9,449 | **,000** |
| a. outcome measure: CATS_Score., dfn = degrees of freedom numerator, dfd degrees of freedom denominator | | | | |

**Estimated marginal means**

| SCCM Level |  |  | Mean difference (T0-T1) | Standarderror (SE) | degrees of freedom (df) | significance (p) | 95% CI for the mean difference | |
| --- | --- | --- | --- | --- | --- | --- | --- | --- |
|  |  |  |  |  |  |  | Lower limit | Upper limit |
| Level 1: |  |  | -,661 | 2,401 | 57,857 | ,784 | -5,467 | 4,146 |
| Level 2: |  |  | -5,430 | 2,436 | 60,907 | **,030** | -10,302 | -,558 |
| Level 3: |  |  | 1,402 | 2,660 | 66,537 | ,600 | -3,908 | 6,712 |
| Level 4: |  |  | 1,380 | 3,192 | 58,413 | ,667 | -5,009 | 7,769 |


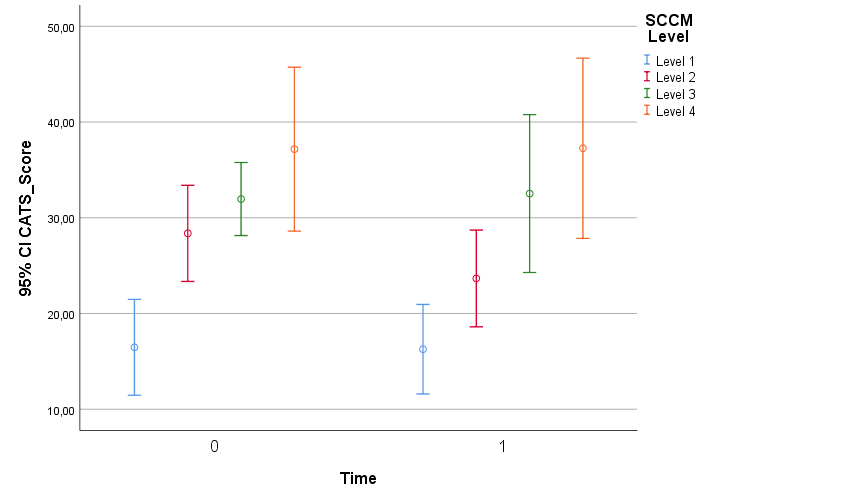

Supplement: Supplementary file 1 — Supplementary file1 (DOCX 40 KB) [file 787_2023_2179_MOESM1_ESM.docx]
